# mTORC2 Activation Mediated by Mesenchymal Stem Cell-Secreted Hepatocyte Growth Factors for the Recovery of Lipopolysaccharide-Induced Vascular Endothelial Barrier

**DOI:** 10.1155/2021/9981589

**Published:** 2021-10-18

**Authors:** Shan-Shan Meng, Feng-Mei Guo, Li-Li Huang, Ying-Zi Huang, Jian-Feng Xie, Cong-Shan Yang, Hai-Bo Qiu, Yi Yang

**Affiliations:** Jiangsu Provincial Key Laboratory of Critical Care Medicine, Department of Critical Care Medicine, Zhongda Hospital, School of Medicine, Southeast University, Nanjing 210009, China

## Abstract

Acute lung injury (ALI)/acute respiratory distress syndrome (ARDS) is characterized by pulmonary microvascular endothelial barrier dysfunction. Mesenchymal stem cell-secreted hepatocyte growth factor (HGF) has positive effects of lipopolysaccharide- (LPS-) induced pulmonary endothelial barrier. Studies have exhibited the mammalian TORC1 (mTORC1) signaling is of potent angiogenesis effects. The mTOR protein kinase has two distinct multiprotein complexes mTORC1 and mTORC2 that regulate different branches of the mTOR network. However, detailed mTORC2 mechanisms of HGF protective effects remain poorly defined. Therefore, the aim of this study was to determine whether mTORC2 mediated protective effects of MSC-secreted HGF against LPS-induced pulmonary microvascular endothelial barrier dysfunction activated like mTORC1 activation. We introduced MSC-PMVEC coculture transwell system and recombinant murine HGF on LPS-induced endothelial cell barrier dysfunction in vitro and then explored potential mechanisms by lentivirus vector-mediated HGF, mTORC1 (raptor), and mTORC2 (rictor) gene knockdown modification. Endothelial paracellular and transcellular permeability, adherent junction protein (VE-Cadherin), cell proliferation, apoptosis, and mTOR-associated proteins were tested. These revealed that HGF could promote quick reestablishment of adherent junction VE-cadherin and decrease endothelial paracellular and transcellular permeability during LSP-induced endothelial dysfunction with the involvement of mTORC2 (rictor) and mTORC1 (raptor) pathways. Raptor and rictor knockdown in LPS-induced PMEVECs with stimulation of HGF increased apoptosis ratio, activated Cleaved-Caspase-3 expression, and downregulated cell proliferation. Moreover, mTORC2/Akt but not mTORC2/PKC had significance on HGF endothelial protective effects. Taken together, these highlight activation mTORC2 pathway could also contribute to vascular endothelial barrier recovery by MSC-secreted HGF in LPS stimulation.

## 1. Introduction

Clinical manifestations of acute lung injury (ALI)/acute respiratory distress syndrome (ARDS) are variable and complex. Pathology mechanisms are the magnitude injury to pulmonary microvascular endothelial barrier [1–2]. The possibility of attenuating lung endothelial barrier dysfunction at early stage is a critical determinant of endothelial recovery. Some researchers have devoted to investigate new approaches to lung endothelial barrier, such as endothelin-1 receptor antagonist, phosphodiesterase inhibitor, and prostaglandin [3]. However, the results of protective effect were not ideal due to complex physiological environments, drug doses, and administration time. Mesenchymal stem cells (MSC), which derived from the early development of mesoderm and ectoderm, have capacity with high self-renewal, multiple differentiation potential, reducing vascular endothelium permeability, and increasing pulmonary water clearance. MSC protective against lung injury has matured gradually with the involvement of MSC paracrine inflammation factors [4–5]. We previously have found that MSC-secreted hepatocyte growth factor (HGF) had positive effects of endothelial barrier recovery in ARDS [6–7]. HGF could elicit potent endothelial barrier protective effects on endothelial cells in vitro and decrease lung tissues injured under ALI conditions in vivo [6–7].

HGF is a growth factor which can be secreted by numerous other cell types, such as alveolar macrophages, hepatocytes, melanocytes, and keratinocytes, and a prosurvival mediator that regulates vascular barrier integrity maintenance and appears at raised concentrations in the lung under pathological conditions such as ALI, sepsis, ventilator-induced lung injury, and lung inflammation. In ARDS patients, neutrophils, macrophages, and fibroblasts can also produce HGF which may enhance the alveolar repair process [8–10]. Moreover, HGF can interact with the target cells via c-Met receptor and c-Met receptor for HGF is highly expressed in lung tissues.

Studies have exhibited that the Akt/mammalian TOR (mTOR) signaling network has potent angiogenesis and endothelial repair effects [11–13]. The central component of the mTOR protein kinase has two distinct multiprotein complexes that regulate different branches of the mTOR network. The mTOR complex 1 (mTORC1) contains mTOR, regulatory-associated protein of mTOR (raptor), DEPTOR, mLST8, and PRAS40. Differently is that the mTOR complex 2 (mTORC2) contains mTOR, rapamycin-insensitive companion of mTOR (rictor), mSIN1, mLST8, DEPTOR, Protor, G*β*L, TTI1, and TEL2. Raptor is specific for mTORC1, and likewise, rictor is specific for mTORC2. Early studies thought mTORC1 was sensitive to rapamycin and otherwise mTORC2 was resistant. Contrarily, new researches revealed that prolonged rapamycin treatment inhibited mTORC2 [14]. mTORC1 regulates cell growth through the phosphorylation of S6K1 and 4E-BP1, and mTORC2 regulates actin cytoskeletal reorganization via prosurvival kinase Akt and PKC by phosphorylating it on S473. Although mLST8 is not absolutely required for mTORC1 activity, mLST8, rictor, and mSIN1 are all required for mTORC2 [15–16]. Studies have demonstrated that mTORC1 activity can represent a key process in promoting angiogenesis [17–18]. However, detailed mTORC2 mechanisms of HGF protective effects are not certain.

The aim of this study was to determine whether protective effects of MSC-secreted HGF against LPS-induced pulmonary microvascular endothelial barrier dysfunction were activated by the mTORC2 pathway. We introduced MSC-PMVEC coculture transwell system and recombinant murine HGF on endothelial cell barrier dysfunction stimulated by gram-negative bacterial pathogen lipopolysaccharide (LPS) in vitro and then explored potential mechanisms by lentivirus vector-mediated HGF, mTORC1 (raptor), and mTORC2 (rictor) gene knockdown modification. This research contributed to our understanding of mTORC2 endothelial protective involvements induced by MSC-secreted HGF in ARDS.

## 2. Materials and Methods

### 2.1. Cell Culture

MSCs derived from normal mouse bone marrow were purchased from Cyagen Biosciences, Inc. (Guangzhou, China), and pulmonary microvascular endothelial cells (PMVECs) were purchased from Shanghai Zhen Biotechnology, Inc. (Shanghai, China). The supplier identified cells according to cell surface phenotypes or multipotency routinely. MSCs/PMVECs were cultured in DMEM/F12 or DMEM containing 10% fetal bovine serum growth medium (Wisent, China) and humidified 5% CO_2_ incubator at 37°C. We changed the culture media every other day, and passage 3-7 cells were used for the experiment.

### 2.2. Gene Modification

Lentivirus vector-mediated HGF overexpression in MSCs and raptor and rictor knockdown in PMVECs were conducted. Passages less than 6 cells were used for these experiments. The HGF gene overexpression was conducted using lentivirus vector, and overexpression specific for EF-1*α*-enhanced green fluorescent protein (EGFP) was used as a negative control. The raptor and rictor knockdown was conducted using lentivirus vector (Raptor-Target-Seq: CCTCATCGTCAAGTCCTTCAA; Rictor-Target-Seq: GCTGAGATTTCTTTCCATTCC), and knockdown specific for EGFP was used as a negative control. The lentivirus was packaged in 293T cells (Cyagen Biosciences) with the aid of three packaging plasmids, and then, a higher titer of lentivirus was obtained. MSCs with HGF overexpression were with transfection and screened by antibiotic blasticidin for 7-14 days. Subsequently, MSCs/PMVECs carrying empty vectors and EGFP (MSC-control and shRNA-control) or MSCs/PMVECs carrying both the target gene and EGFP (MSC-HGF, shRaptor, and shRictor) were harvested and tested transfection efficiency by real-time quantitative polymerase chain reaction and expression efficiency by a fluorescence microscope.

### 2.3. Coculture Transwell System and Reagent Treatments

A transwell coculture system was used to investigate MSC paracrine protective effects on endothelial barrier. MSCs with or without overexpression HGF gene were seeded to upper transwell chamber (0.4 *μ*m pore size polyester membrane from Corning, Inc., USA), and PMVECs were seeded to lower chamber (six-well culture plates). We cultured for 1-3 days to allow the growth of a confluent monolayer and then added LPS (100 ng/ml, Sigma, USA) to PMVECs.

Gram-negative bacterial pathogen lipopolysaccharide (LPS, 100 ng/ml, Sigma, USA) was treated with PMVECs to mimic endothelial barrier dysfunction. To determine the roles and mechanisms HGF, recombinant murine HGF (20 ng/ml, ProSpec, Israel) was introduced to LPS-induced PMVECs. Doses of LPS and HGF were applied according to our preliminary experiments. Moreover, PBS was applied as negative control and Akt inhibitor AZD53631 (1 *μ*M, Selleck, USA) or PKC inhibitor enzastaurin (LY317615) (1 *μ*M, Selleck) was applied to inhibit the activation in PMVECs. Phosphatidylinositol 3,4,5-trisphosphate as PI3K facilitates (PtdIns(3,4,5)P3, 25 ng/ml, Sigma) was also used to active mTORC2 signaling [19].

### 2.4. Endothelial Permeability Measurement

A transwell cultivate system was applied to endothelial permeability measurement. We seeded PMVECs to the upper chamber (0.4 *μ*m pore size polyester membrane from Corning, Inc., USA), cultured for 1-3 days for the growth of a confluent monolayer, and then stimulated endothelium with different drug treatments. Detailed methods were tested as described previously [20]. We added 10 *μ*l Alexa Fluor 647 labeled-dextran (Thermo Fisher Scientific, USA) or Alexa Fluor 647 labeled-BSA (Nanocs, USA) to the upper chamber in 37°C condition for 40 min to relatively test endothelial paracellular and transcellular permeability. Finally, 100 *μ*l upper or lower medium was withdrawn and observed by fluorescence intensity at 650 nm excitation and 668 nm emission wavelengths with a microplate reader.

### 2.5. Western Blot (WB) Analysis

Cell total proteins were split using RIPA lysis buffer supplemented with 1 mmol/l phenylmethanesulfonyl fluoride (Beyotime) and separated with SDS-PAGE condensed electrophoresis (Beyotime). Polyvinylidene fluoride membrane (Beyotime) was cut off according to a size of gel, arrange gel-membrane sandwich and use it in electrotransfer. Next, the membranes were blocked in 5% BSA for 1 hour at room temperature. Then, primary antibodies against raptor (1 : 1000; Cell Signaling Technology, USA), rictor (1 : 1000; Cell Signaling Technology), VE-cadherin (1 : 1000; abcam, USA), Caspase-3 (1 : 1000; Cell Signaling Technology), Cleaved-Caspase-3 (1 : 1000; Cell Signaling Technology), phosphorylation or total Akt (Ser473) (1 : 1000; Cell Signaling Technology), mTOR (Ser2448) (1 : 1000; Cell Signaling Technology), p70 S6 kinase (p70S6K; Thr389) (1 : 1000; Cell Signaling Technology), PKC-a (Ser657) (1 : 1000; Cell Signaling Technology), and *β*-Actin (1 : 1000; Cell Signaling Technology) were applied with appropriate dilution at 4°C overnight. Peroxidase-conjugated secondary antibody (1 : 3000; Fcmacs) was incubated on the membrane for 1 h at room temperature. Finally, ECL detection was used and membranes were exposed with a chemiluminescence imaging system (Bioshine ChemiQ 4800mini, Ouxiang, Shanghai, China).

### 2.6. Real-Time Quantitative Polymerase Chain Reaction (RT-qPCR)

Total RNA from PMVECs were isolated with TriPure Isolation Reagent (Roche, Switzerland), concentrated with a microplate reader (Infinite M200 Pro, Tecan, Switzerland), and reverse transcriptase was applied (Thermo Fisher Scientific, USA) for cDNA synthesis. We used Primer Express software (Vector NTI advance10) to design specific primer pairs. The following primers were used: *β*-Actin, sense 5′-AGGTCTTTACGGATGTCAACG-3′ and antisense 5′-TCTTTTCCAGCCTTCCTTCTT-3′; raptor, sense 5′-TCTACGACAGGAGGATGGCA-3′ and antisense 5′-ACTGGTTCATGGAGCCACAG-3′; rictor, sense 5′-TATGACCGACCTGGACCCAT-3′ and antisense 5′-CTGTGCTGAGGAGCTTGTGA-3′. Real-Time PCR System (Applied Biosystems, USA) was used to detect the PCR product caused by the binding of SYBR Green (Thermo Fisher Scientific) to dsDNA. The threshold cycle (CT) of each target product is associated with the amplification plot of *β*-Actin. RNA level from target gene is described as gene expression. 2^-△△CT^ is calculated by the distinction of CT values between different groups for relative gene expression.

### 2.7. Flow Cytometry for VE-Cadherin Expression

VE-cadherin expression in PMVECs was analyzed by flow cytometry (ACEA NovoCyte, China) with different drug treatments. For surface staining, primary anti-VE-cadherin antibody (1 : 400; abcam) was added at room temperature for 30 min in the dark. Then, we centrifuged PMVECs and stained these with Alexa Fluor 647-labeled IgG Cross-Adsorbed Secondary antibody (1 : 200; Thermo Fisher Scientific). Results were analyzed by flow cytometer software (ACEA NovoExpress, China).

### 2.8. Cell Viability Assays

PMVECs were seeded into 96-well plates and stimulated with different drugs. Cell viability was performed using Cell Counting Kit-8 (Beyotime, Shanghai, China) according to manufacture instruments. Absorbance was read with a 450 nm wavelength microplate reader.

### 2.9. Cell Apoptosis

Cell apoptosis was determined by Annexin V-PE/7-AAD stained flow cytometry (BD Biosciences, USA). PMVECs were harvested and resuspended in 1x binding buffer at a density of 5.0 × 10^5^ cells/ml. 5 *μ*l of phycoerythrin- (PE-) conjugated Annexin V (BD) and 5 *μ*l of 7-Aminoactinomycin D (7-AAD, BD) were incubated with one hundred *μ*l of the binding buffer at room temperature (25°C) in the dark for 15 min. Finally, samples were analyzed by a flow cytometer (ACEA NovoCyte). Early apoptotic (PE positive, 7-AAD negative), late apoptotic, and dead cells (PE positive, 7-AAD positive) can be separated on the basis of a double-labeling for Annexin V-PE and 7-AAD.

### 2.10. Statistical Analyses

Results in this paper were performed with GraphPad Prism 7.0 software and displayed as the mean ± standard deviation. Tukey's multiple comparison tests, one-way analysis of variance, and Student's *t*-test were applied to statistical analysis. *p* value < 0.05 was considered as significant statistic differences.

## 3. Results

### 3.1. MSC-Secreted HGF Decreases LPS-Induced Pulmonary Microvascular Endothelial Cell (PMVEC) Barrier Dysfunction

**Figure 1:**
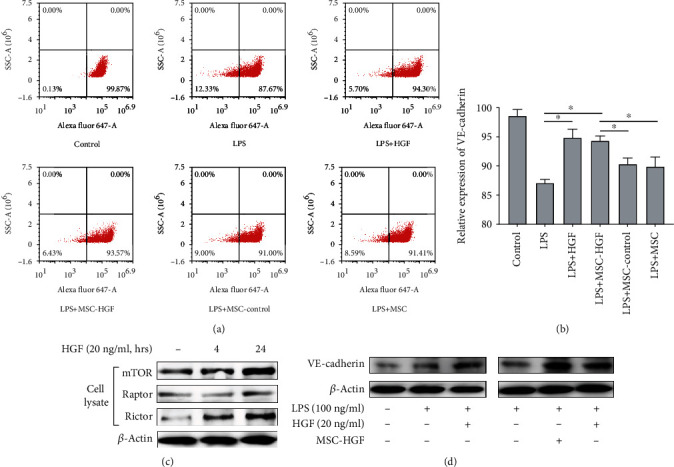
Effects of MSC-secreted HGF on LPS-induced PMVEC barrier. We used 24 h transwell coculture transwell system for culturing PMVECs with MSC, overexpression HGF of MSC (MSC-HGF), or HGF negative control of MSC (MSC-control). Moreover, PMVECs were treated with HGF (20 ng/ml), with or without stimulation with LPS (100 ng/ml) for 4 h and 24 h. (a) Flow cytometry scatter plot of 4 h VE-cadherin expression with MSC-secreted HGF in LPS-induced PMVECs. (b) Flow cytometry cell counts (%) of 4 h VE-cadherin expression with MSC-secreted HGF in LPS-induced PMVECs. (c) The effect of HGF (20 ng/ml) to LPS-induced PMVEC endothelium lysate tested by western blot with 0.4 and 24 h. (d) VE-cadherin protein expression change with MSC-secreted HGF in LPS-induced PMVECs tested by western blot with 24 h. Results are mean ± SD (*n* = 3). ^∗^*p* < 0.05.

We firstly evaluated the effects of MSC-secreted HGF on LPS-induced PMVEC barrier. VE-cadherin, an important adherent junction glycoprotein, was tested by a flow cytometer and western blot with 4 h and 24 h treatment. The coculture system test demonstrated that LPS-stimulated endothelial barrier with MSC overexpression HGF increased adherent junction protein VE-cadherin with examination of flow cytometry and western blot (Figures 1(a), 1(b), and 1(d)). Similarly, recombinant HGF also increased VE-cadherin expression (Figures 1(a), 1(b), and 1(d)). Moreover, cell lysate displayed that mTOR, raptor, and rictor were activated with prolonged treatments of HGF (Figure 1(c)). These suggested that HGF could promote quick reestablishment of adherent junction during LSP-induced endothelial injury and mTOR might contribute to it.

### 3.2. mTORC2 Like mTORC1 Can Also Be Activated by HGF in LPS-Stimulated PMVECs

**Figure 2:**
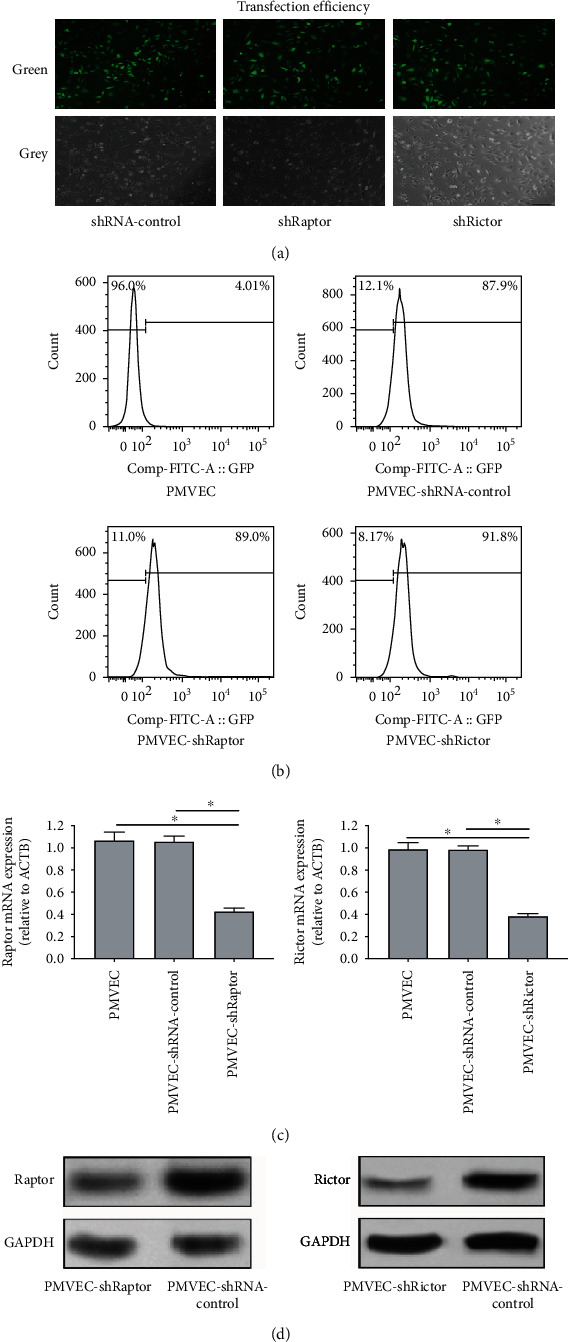
Conduction of lentivirus vector-mediated mTORC1 (raptor) and mTORC2 (rictor) knockdown in PMVECs. (a) Transfection efficiency of shRaptor and shRictor in PMVECs with a fluorescence microscope (×200). (b) Transfection efficiency of shRaptor and shRictor in PMVECs with flow cytometry analysis. (c) RT-PCR analysis for relative raptor and rictor mRNA expression in PMVECs with gene knockdown. (d) WB analysis for relative raptor and rictor protein level in PMVECs with gene knockdown. Results are mean ± SD (*n* = 3). ^∗^*p* < 0.05.

**Figure 3:**
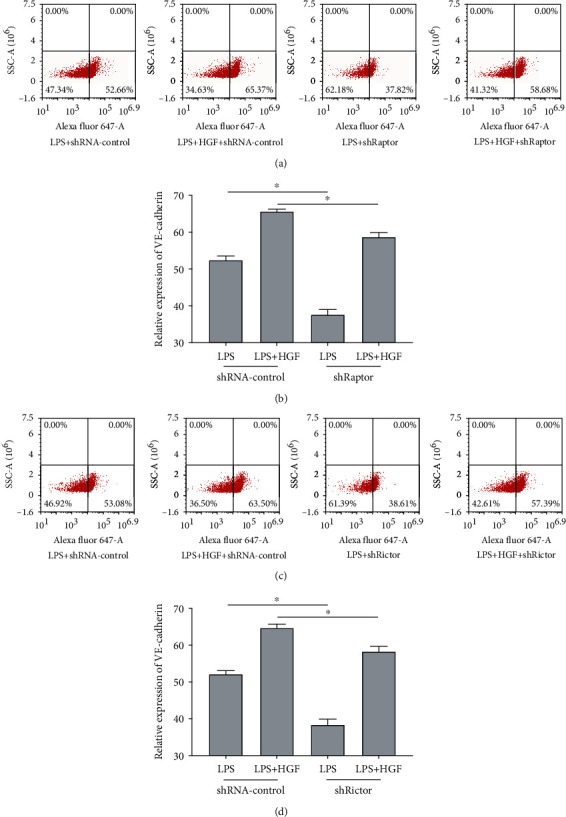
Effects of mTORC1 and mTORC2 to HGF on VE-cadherin expression in LPS-induced PMVEC barrier. Lentivirus vector-mediated raptor and rictor knockdown in PMVECs (shRaptor and shRictor as knockdown, shRNA-control as negative control) was conducted. PMVECs were treated with HGF (20 ng/ml), with or without stimulation with LPS (100 ng/ml) for 4 h. (a) Flow cytometry scatter plot of 4 h VE-cadherin expression with HGF in LPS-induced PMVEC-shRaptor. (b) Flow cytometry cell counts (%) of 4 h VE-cadherin expression with HGF in LPS-induced PMVEC-shRaptor. (c) Flow cytometry scatter plot of 4 h VE-cadherin expression with HGF in LPS-induced PMVEC-shRictor. (d) Flow cytometry cell counts (%) of 4 h VE-cadherin expression with HGF in LPS-induced PMVEC-shRictor. Results are mean ± SD (*n* = 3). ^∗^*p* < 0.05.

**Figure 4:**
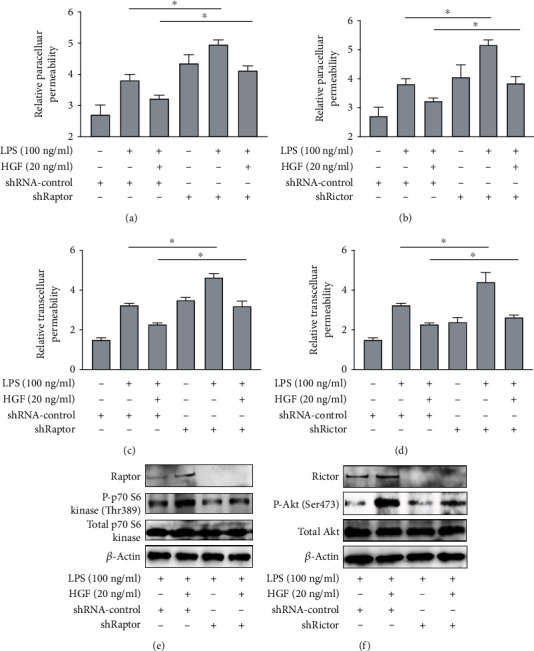
Effects of mTORC1 and mTORC2 to HGF on endothelial permeability in LPS-induced PMVECs. Lentivirus vector-mediated mTORC1 (raptor) and mTORC2 (rictor) knockdown in PMVECs (shRaptor and shRictor as knockdown, shRNA-control as negative control) was conducted. PMVECs were treated with HGF (20 ng/ml), with or without stimulation with LPS (100 ng/ml) for 24 h. (a) The effects of mTORC1 to relative paracellular permeability of HGF on LPS-induced PMVEC permeability with Alexa Fluor 647-dextran. (b) The effects of mTORC2 to relative paracellular permeability of HGF on LPS-induced PMVEC permeability with Alexa Fluor 647-dextran. (c) The effects of mTORC1 to relative transcellular permeability of HGF on LPS-induced PMVEC permeability with Alexa Fluor 647-BSA. (d) The effects of mTORC2 to relative transcellular permeability of HGF on LPS-induced PMVEC permeability with Alexa Fluor 647-BSA. (e) Evaluation of HGF on mTORC1 signaling pathway for western blot. (f) Evaluation of HGF on mTORC2 signaling pathway for western blot. Results are mean ± SD (*n* = 3). ^∗^*p* < 0.05.

mTOR consists of two distinct complexes, and more attention has been paid to mTORC1. mTORC2 detailed function is still not clear. Thus, we continually observed whether mTORC2 mediated HGF protective endothelial effects and compared it with mTORC1 (raptor). First, lentivirus vector-mediated mTORC1 (raptor) and mTORC2 (rictor) knockdown in PMVECs (shRaptor and shRictor as a knockdown group, shRNA-control as negative control) was conducted (Figure 2). The transduction efficacy in both the infections was above 90% (Figures 2(a) and 2(b)). The RT-PCR test demonstrated approximately 60% lower raptor and rictor mRNA expression in PMVEC-shRaptor/PMVEC-shRictor than shRNA-control (Figure 2(c)). WB analysis showed that lower raptor and rictor protein was expressed in PMVEC-shRaptor/PMVEC-shRictor than shRNA-control (Figure 2(d)). PMVECs were treated with HGF (20 ng/ml), with or without stimulation with LPS (100 ng/ml) for 24 h. To evaluate mTORC2 signaling pathway protective effects, we tested mTORC2 as well as mTORC1 signaling and made comparison between them. Flow cytometry gives results that adherent junction protein VE-cadherin could be examined downregulated in raptor and rictor knockdown even with the protective factor HGF in 2 h early stage (Figure 3). The reflection of endothelial damage was so quick, and two hours were enough. Interestingly, further data displayed that shRaptor and shRictor raised effects paracellular and transcellular permeability of HGF decreasing on LPS-induced PMVEC permeability with Alexa Fluor 647-dextran (Figures 4(a) and 4(b)) and Alexa Fluor 647-BSA (Figures 4(c) and 4(d)) in 24 h. WB analysis showed that phosphorylation protein level change with HGF treatment. HGF promoted raptor and phosphorylation p70S6K (Thr389) protein levels in LPS-induced PMVECs, and shRaptor reversed the activation progress (Figure 4(e)). Diversely, HGF increased rictor and downstream target of phosphorylation Akt (Ser473) protein levels in LPS-induced PMVECs. Rictor knockdown in PMVECs reversed HGF activation effects (Figure 4(f)). These might be explained by that VE-cadherin loss might lead to enlarged intercellular space and endothelial permeability. Collectively, mTORC2 core protein rictor could also be activated like mTORC1 in adherent junction remodeling and endothelial barrier recovery.

### 3.3. HGF Protects LPS-Stimulated PMVEC Barrier through mTORC2/Akt but Not mTORC2/PKC

**Figure 5:**
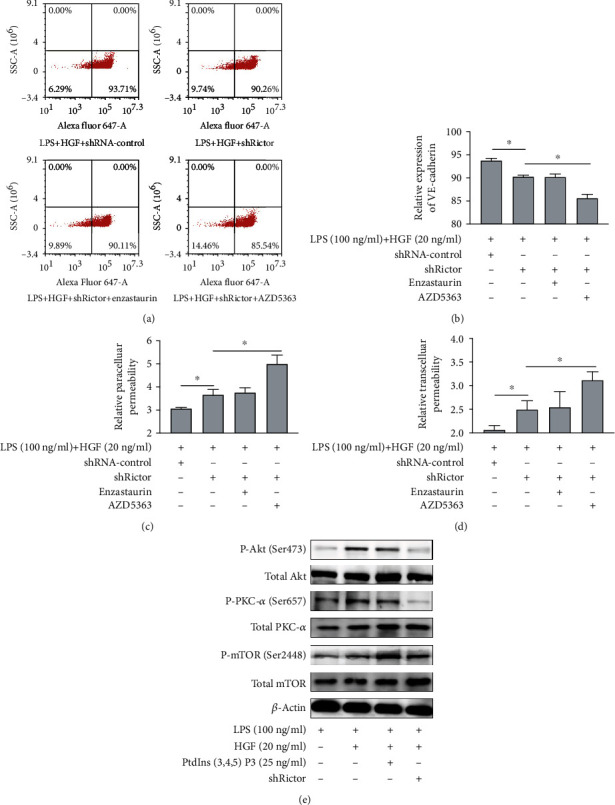
Effects of mTORC2/Akt and mTORC2/PKC signaling to HGF on endothelial barrier in LPS-induced PMVECs. PMVECs were treated with HGF (20 ng/ml), with or without stimulation with LPS (100 ng/ml) for 24 h. And enzastaurin (2 *μ*M) and AZD5363 (1 *μ*M) were, respectively, used as PKC and Akt inhibitors. PtdIns(3,4,5)P3 (25ng/ml) was used to active mTORC2 as positive control. (a) Flow cytometry scatter plot of 4 h VE-cadherin expression with HGF in LPS-induced PMVECs under Akt and PKC inhibition. (b) Flow cytometry cell counts (%) of 4 h VE-cadherin expression with HGF in LPS-induced PMVECs under Akt and PKC inhibition. (c) The effects of Akt and PKC inhibitors to relative paracellular permeability of HGF on LPS-induced PMVEC permeability with Alexa Fluor 647-dextran. (d) The effects of Akt and PKC inhibitors to relative transcellular permeability of HGF on LPS-induced PMVEC permeability with Alexa Fluor 647-BSA. Results are mean ± SD (*n* = 3). ^∗^*p* < 0.05. (e) Evaluation of HGF on the mTORC signaling pathway for western blot.

The following experiments examined the role of mTORC2/Akt and mTORC2/PKC signaling mediating protective effects of HGF on LPS-stimulated endothelial barrier. PMVEC-shRictor were preincubated with Akt inhibitor AZD5363 (1 *μ*M) or PKC inhibitor enzastaurin (2 *μ*M) and stimulated with LPS and HGF. It dramatically accelerated barrier dysfunction in Akt inhibitor AZD5363 stimulation rather than PKC inhibitor enzastaurin (Figure 5). HGF protective effects of VE-cadherin expression in LPS-induced PMVEC-shRictor staining with Alexa Fluor 647 were inhibited by AZD5363 rather than enzastaurin (Figures 5(a) and 5(b)). And inhibition of Akt decreased the protective effect of HGF on endothelial paracellular (Figure 5(c)) and transcellular (Figure 5(d)) permeability. PKC inhibitor enzastaurin function did not have remarkable change (Figures 5(c) and 5(d)). Furthermore, we used PtdIns(3,4,5)P3 to activate mTORC2 as positive control (Figure 5(e)). HGF could promote phosphorylation level of mTOR (Ser2448) and Akt (Ser473) other than PKC-*α* (Ser657). In all, mTORC2/Akt but not mTORC2/PKC had significance on HGF endothelial protective effects.

### 3.4. HGF Improves Cell Proliferation and Attenuated Cell Apoptosis via mTORC1 and mTORC2

**Figure 6:**
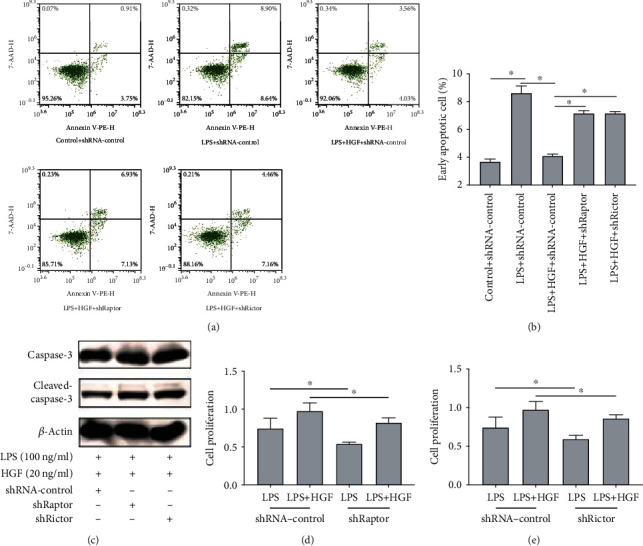
Effects of mTORC1 and mTORC2 to HGF on endothelial proliferation and apoptosis in LPS-induced PMVECs. Lentivirus vector-mediated mTORC1 and mTORC2 (raptor and rictor) knockdown in PMVECs (shRaptor and shRictor as knockdown, shRNA-control as negative control) was conducted. PMVECs were treated with HGF (20 ng/ml), with or without stimulation with LPS (100 ng/ml) for 24 h. (a) Flow-based scatter diagram of mTORC1/mTORC2 to cell apoptosis of HGF on LPS-induced PMVECs. (b) Flow-based early apoptosis ratio of mTORC1/mTORC2 to cell apoptosis of HGF on LPS-induced PMVECs. (c) The effects of mTORC1 and mTORC2 to apoptosis-associated Caspase-3 protein expression of LPS-induced PMVECs with HGF treatments tested by western blot. (d) The effects of mTORC1 to cell proliferation of HGF on LPS-induced PMVECs with CCK8. (e) The effects of mTORC2 to cell proliferation of HGF on LPS-induced PMVECs with CCK8. Results are mean ± SD (*n* = 3). ^∗^*p* < 0.05.

Finally, we evaluated PMVEC surviving capacity. Surviving capacity means proliferation capacity and apoptosis, which were, respectively, tested by CCK8 reagent, Annexin V-PE/7-AAD stained flow cytometry, and Caspase-3 protein analysis. PMVECs were treated with HGF (20 ng/ml), with or without stimulation with LPS (100 ng/ml) for 4 h. We used shRaptor and shRictor PMVEC to mimic mTORC1 and mTORC2 inhibition. The data revealed that HGF attenuated cell apoptosis and raised cell proliferation (Figure 6). Raptor and rictor knockdown reverses the results (Figure 6). Annexin V-PE/7-AAD stained flow cytometry reflected early apoptosis level of PMVECs. Raptor and rictor knockdown increased the Annexin V-PE(+)/7-AAD(-) ratio (Figures 6(a) and 6(b)). Apoptosis critical gene Caspase-3 was also activated to Cleaved-Caspase-3 when raptor and rictor were knocked down (Figure 6(c)). CCK8 reagent showed that HGF could improve cell proliferation induced by LPS and abrogated by raptor and rictor knockdown (Figures 6(d) and 6(e)). These implied that HGF have the effects of cell proliferation and apoptosis via mTORC1 and mTORC2.

## 4. Discussion

Our study revealed mTORC2 like mTORC1 as an important signaling of regulation of MSC-secreted HGF protective against LPS-induced lung endothelial dysfunction. mTORC1 and mTORC2 are two functionally distinct serine/threonine kinase complexes that share the same subunit mTOR conserved in slime mold, files, and mammals [21]. mTORC1 regulates protein synthesis, cell growth, and autophagy [22–23]. mTORC1 appears to be specifically involved in angiogenesis [18]. Arginase-II could activate mTORC1 in vascular cell senescence and apoptosis [17]. mTORC2 coordinated pulmonary artery smooth muscle cell metabolism, proliferation, and survival in pulmonary arterial hypertension [24]. Although above studies suggest mTORC1 inactivation leading to vascular survival and angiogenesis, roles of mTORC2 activation of HGF protective endothelial effects in ARDS remain elusive. In view of this, the role of mTORC2 was investigated in pulmonary endothelial barrier particularly to MSC-secreted HGF. Overexpression HGF of MSC and PMVEC co-culture system were applied to observe MSC-secreted HGF protective effects in endothelial recovery function. It also revealed that both endothelial barrier and survival markers specific to MSC-secreted HGF were downregulated due to rictor and raptor deletion which are approaches to abrogate mTORC2 activation like mTORC1. What is more, HGF protect endothelial effects via mTORC2/Akt not mTORC2/PKC. Endothelial protective effects mediated by the mTORC2 pathway were verified in our experiments.

Microvascular endothelial barrier dysfunction refers to endothelial permeability and apoptosis. Endothelial permeability dysfunction is considered as transcellular and paracellular dysfunction. Endothelial connection is the core of endothelial paracellular permeability and VE-cadherin-based adherent junction block water and solute flow and ensures the stability of internal environment. Blocking antibodies to VE-cadherin increased monolayer permeability in cultured cells [25]. mTORC2 integrity requires rictor, SIN1, and mLST8, and any subunit deficiency is sufficient to downregulate and ablate the mTORC2 activity towards Akt and PKC. Knockdown rictor is regarded as mTORC2 deficiency activity. mTORC2 and rictor have been implicated in endothelial barrier damage diseases [26–27] and play essential roles in embryonic growth and actin cytoskeleton organization [28]. Raptor, which is essential for mTORC1 activity, is interacted with mTOR activity and contains docking sites for the mTOR substrates, 4E-BP and S6K1 [29]. Growth factors and insulin activate mTORC1 via the activation of phosphatidylinositol 3-OH kinase (PI3K) and its downstream effector Akt and 4E-BP1 and S6K1 activation. Based on these, we used raptor in mTORC1 detection and mTORC1 could mediate HGF endothelial protection. We further made rictor detection to verify if endothelial permeability and adherent junction remodeling was also dependent of mTORC2 integrity. Similarly, permeability analysis and the VE-cadherin test displayed that mTORC2 contributed to HGF protective effects against LPS-induced endothelial barrier dysfunction. This revealed mTORC2 contributed to HGF protective effects of endothelial barrier.

mTORC2 regulates cellular proliferation and metabolism, in part through the regulation of Akt, PKC, IGF-IR, InsR, and SGK-1. Growth factors regulated mTORC2 activation and the activity of Akt and PKC [30]. Further experiments were carried out to ascertain the definitive role of mTORC2/Akt and mTORC2/PKC in HGF endothelial protective effects. Interestingly, we found that Akt inhibitor not PKC inhibitor attenuated HGF endothelial protective effects of raising VE-cadherin and decreasing endothelial permeability. What is more, previous evidences were found for mTORC2 direct nonspecific activation by phosphatidylinositol 3,4,5-trisphosphate as PI3K facilitates (PtdIns(3,4,5)P3) [19]. Hence, we used PtdIns(3,4,5)P3 as HGF positive control. HGF could promote the phosphorylation level of mTOR (Ser2448) and Akt (Ser473) other than PKC-*α* (Ser657). These displayed that HGF-induced protective endothelial barrier was precisely mediated by mTORC2/Akt axis rather than mTORC2/PKC.

As described above, mTORC2 function is placed upstream of Akt and mTORC1, whereas mTORC1 is placed both downstream and upstream of Akt. Considering mTOR is the only important subunit shared by mTORC1 and mTORC2, the two complex normal competition exists because of mTORC1 and mTORC2 integrity. If one of these two structurally and functionally changes, the incorporation of mTOR into one complex would limit its access to the other. In our study, we found that knockdown of rictor could enhance mTORC1 activity. It may have a certain level of equilibrium between mTORC1 and mTORC2 competing for mTOR.

There are some limitations in our experiments. Our study only focuses on the effects of MSC-secreted HGF; other growth factor effects were not certain. Moreover, it is just a cell experiment; more in vivo studies should be investigated in further experiments.

## 5. Conclusions

Hence, activation of raptor and rictor pathways are involved in MSC-secreted HGF protective of LPS-induced endothelial barrier reestablishment. These findings provide new insights into endothelial recovery regulation in lung tissue. As reviewed by this study, it is emphasized that mTORC2 like mTORC1 is an important target in HGF protective effects of endothelial barrier in LPS stimulation.

## Data Availability

The data used to support the findings of this study are included within the article.

## Abbreviations

ALI: Acute lung injury
ARDS: Acute respiratory distress syndrome
CCK-8: Cell Counting Kit-8
HGF: Hepatocyte growth factor
LPS: Lipopolysaccharide
MSCs: Mesenchymal stem cells
mTOR: Mammalian TOR
mTORC1: mTOR complex 1
mTORC2: mTOR complex 2
PE: Phycoerythrin
PMVEC: Pulmonary microvascular endothelial cell
WB: Western blot
RT-qPCR: Real-time quantitative polymerase chain reaction
7-AAD: 7-Aminoactinomycin D.
